# Dogs with idiopathic epilepsy and focal epileptic seizures lack hilar neuron loss and mossy fiber sprouting of temporal lobe epilepsy

**DOI:** 10.2460/ajvr.25.07.0260

**Published:** 2026-01-23

**Authors:** Kristin Baert, Sue Osting, Elizabeth DiPaola, Paul Buckmaster, Starr Cameron

**Affiliations:** 1 Department of Medical Sciences, School of Veterinary Medicine, University of Wisconsin-Madison, Madison, WI; 2 Department of Neurology, School of Medicine and Public Health, University of Wisconsin-Madison, Madison, WI; 3 BluePearl Veterinary Hospital, Malvern, PA; 4 Department of Comparative Medicine, Stanford University, Stanford, CA; 5 Department of Neurology & Neurosurgical Sciences, Stanford University, Stanford, CA

**Keywords:** partial seizures, canine, hippocampal sclerosis, Timm stain, genetic epilepsy

## Abstract

**Objective:**

To evaluate neuropathological changes in dogs with idiopathic epilepsy and history of focal seizures using previously described criteria for temporal lobe epilepsy (TLE) in humans and other animals.

**Methods:**

This was a prospective descriptive study. Dogs of any age, sex, or breed with a history of idiopathic epilepsy and focal seizures were included. Immediately following euthanasia, dogs were perfused, and tissue was collected. Brains were sectioned, mounted, and stained. Nissl-stained slides were used for stereology, and Timm stain was used to evaluate for evidence of mossy fiber sprouting.

**Results:**

7 dogs were included in the final analysis. For stereological cell counts evaluating hilar neuron loss, no case was significantly lower in number of neurons. Additional analysis compared right and left to look for asymmetry, and none were significant (*P* = .259). Using Timm stain to evaluate mossy fiber sprouting, no cases showed extensive sprouting, and no asymmetry was detected between left and right hippocampi (*P* = .054).

**Conclusions:**

In our cohort, while there were changes associated with seizures detected within the hippocampi, none were severe or consistent enough to qualify as TLE using the human criteria.

**Clinical Relevance:**

Our study focused on dogs with focal seizures as their main seizure presentation as that is the most common semiology in people with TLE. However, a future study could consider evaluating the neuropathology of dogs with asymmetric hippocampi detected on MRI. If TLE can be shown in dogs, then advanced treatment options, such as surgery or laser ablation, could be considered in refractory patients.

Epilepsy is the most common chronic neurological condition diagnosed and treated in dogs, and estimates of prevalence range from 0.6% to 0.75%.^1^ In dogs, epilepsy is classified as idiopathic or structural in origin. Idiopathic epilepsy (IE) is commonly a diagnosis of exclusion in clinical practice, and a genetic influence for many breeds with IE is supported.^2^ While there is breed-specific variation in seizure semiology, generalized seizures or focal seizures progressing to generalized seizures tend to be the most common semiology in dogs with IE.^1^ In humans, some cases of IE have no neuropathologic abnormalities, but mesial temporal sclerosis is a common finding.^3^ Structural epilepsy (SE) comprises several different etiologies in dogs, including intracranial neoplasia, meningoencephalitis of unknown etiology, traumatic brain injury, cerebrovascular accidents, congenital malformation, degenerative storage diseases, and intracranial infectious diseases. In SE in dogs, generalized seizures are more common than focal seizures, and both older age and the presence of interictal abnormalities are associated with an increased prevalence of SE compared to IE.^4^ Intracranial neoplasia is usually easily identified grossly or neuropathologically, and other causes of SE may show changes such as gliosis and neuroinflammation.^3^

In people with epilepsy, the most common cause is temporal lobe epilepsy (TLE),^5^ which is a well-defined disorder in humans in clinical presentation (semiology) and neuropathology. The most common semiology is with automatisms, which can present as chewing, lip smacking, swallowing, or fumbling of the hands^6^ (ie, focal epileptic seizures). Hippocampal sclerosis (HS) is commonly associated with TLE. Hippocampal sclerosis involves specific patterns of neuronal loss and gliosis and granule cell axon (mossy fiber) sprouting. Not all cases of TLE have mossy fiber sprouting,^7^ but when present, mossy fiber sprouting can distinguish TLE from other causes of HS.^8^ Mossy fiber sprouting and HS in TLE are consistent between humans and mouse models.^9^ Up to 90% of cases of TLE in humans have been determined to have asymmetric hippocampal involvement.^10^

Many people with TLE are refractory to medications.^11^ In these refractory patients, advanced treatment options, such as surgical removal or laser ablation of the affected hippocampus, have been performed. In 58% of cases treated with laser ablation,^12^ patients became seizure-free long term, leading to a significant improvement in quality of life.^13^ Surgery provides an even higher success rate, with approximately 90% of patients becoming seizure-free or experiencing rare seizures after surgery.^14^

Previous work tested whether 7 dogs with idiopathic and medically intractable epilepsy had TLE.^15^ Stereologic cell counting and somatostatin immunoreactivity were performed within the hilus region of the dentate gyrus of the hippocampus as well as Timm staining for mossy fiber sprouting. No evidence of HS consistent with TLE was found. However, most of this small cohort of dogs presented with generalized seizures and not focal seizures. As focal epileptic seizures are the most common semiology in people with TLE, the population of the previous study was not representative of dogs likely to have TLE. Additionally, recent evidence from MRI studies^16^ has found hippocampal asymmetry in dogs with presumptive IE. Therefore, neuropathological investigation into the pattern of neuronal changes in dogs with focal epileptic seizures is necessary to determine if TLE occurs in dogs.

We hypothesized that a subset of epileptic dogs with focal epileptic seizures would have neuropathological changes consistent with what has been described in people and other species as TLE. Neuron loss in the hilus of the dentate gyrus can be the only obvious cell loss in people with TLE,^17,18^ carnivores,^19^ and rodents.^20^ This study examined the extent of hilar neuron loss and mossy fiber sprouting within the dentate gyrus in dogs with IE, specifically with a history of focal epileptic seizures.

## Methods

This study protocol was approved by the IACUC at the University of Wisconsin-Madison School of Veterinary Medicine (No. V006178-R01).

### Study population

Dogs previously diagnosed with tier 2 IE based on the International Veterinary Epilepsy Task Force classification guidelines^2^ with a history of focal epileptic seizures of any age, sex, breed, or weight were recruited for the study. Dogs with any other known or suspected intracranial disease were excluded from participating in this study. Additionally, the main semiology of the patient’s seizures needed to be either focal epileptic seizures or focal onset with secondary generalization. Dogs with only or primarily generalized seizures were excluded from this study.

### Perfusion and tissue preparation

Following euthanasia by IV pentobarbital at the University of Wisconsin-Madison Veterinary Care for reasons not related to this study, and with the consent of the owners, dogs were perfused at 0.2 L/min from the ascending aorta for 2 minutes with 0.9% sodium chloride, 5 minutes with 0.37% sodium sulfide, another 1 minute with 0.9% sodium chloride, and then approximately 20 minutes with 4% formaldehyde in 0.1M phosphate buffer. Brains were removed and placed in a 4% paraformaldehyde solution for 1 week, placed in a vacuum-sealed pouch in Fluorinert, an electron-neutral liquid, and then imaged using diffusion tensor imaging MRI at the Wisconsin Institute for Medical Research using a 4.7-Tesla small-animal scanner (Agilent Technologies). Following imaging, the brains were placed in a 30% sucrose, 4% paraformaldehyde solution and stored at 4 °C for approximately 7 days or until the brain sank. Brains were sectioned into 4 blocks, including rostral, caudal, right middle, and left middle sections, and were then snap frozen in 2-methylbutane with dry ice for 10 to 15 minutes. Brain blocks were stored wrapped in aluminum foil in plastic boxes at −80 °C until sectioning was performed. The brains were sectioned using a slide microtome set at 40 μm, and transverse sections were stored at −80 °C until being mounted.

Sections were mounted, cover slipped, and processed for either Nissl staining or Timm staining at 18-section intervals. Both the right and left hippocampi were processed for each dog as the neuropathology of human TLE is frequently lateralized.^21^

### Tissue analysis

**Figure 1—:**
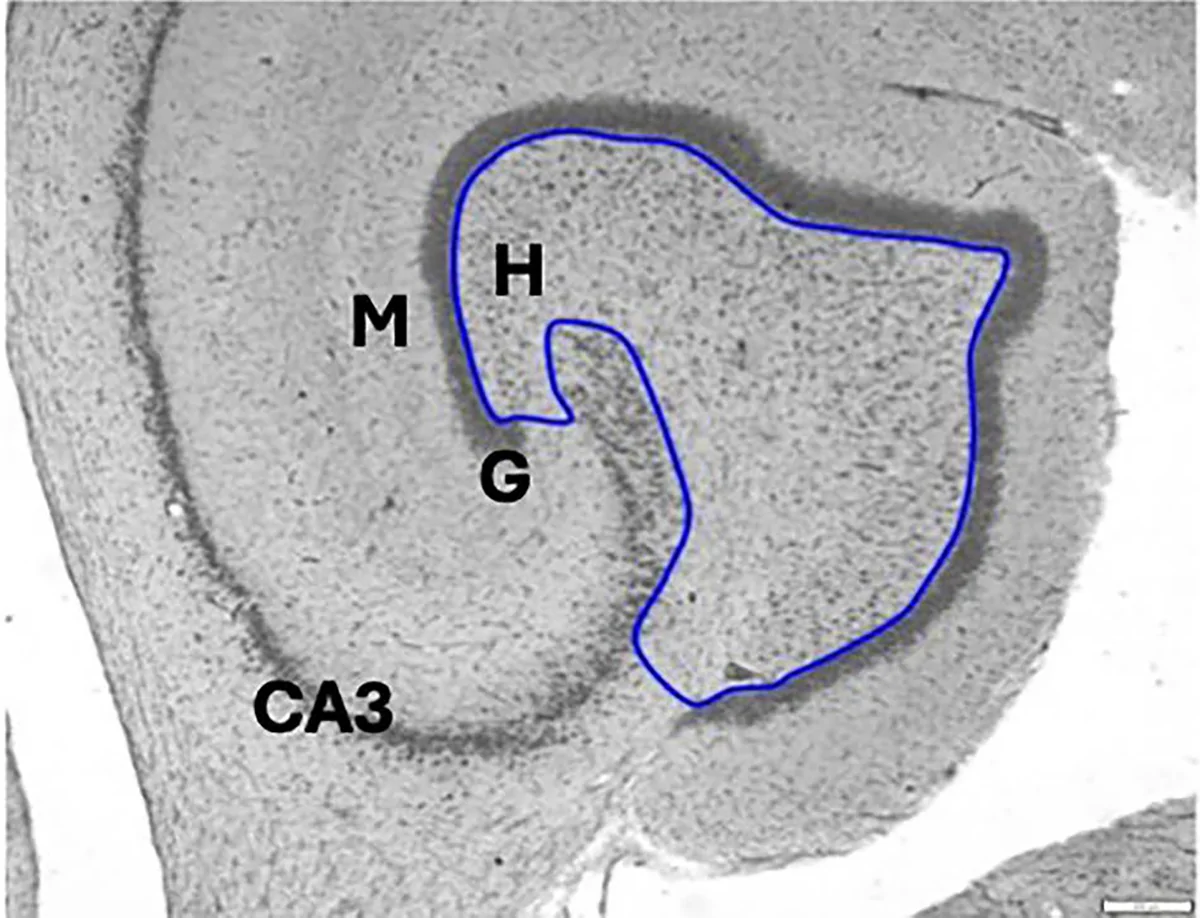
Nissl-stained hippocampus at 40X total magnification, with the blue outline indicating the hilus. CA3 = Proximal cornu ammonis 3 pyramidal cell layer. G = Granule cell layer. H = Hilus. M = Molecular layer. Scale bar = 200 μm.

Nissl-stained neurons in the hilus of each hippocampus were examined with a microscope equipped with a camera, motorized stage, and StereoInvestigator software (MBF Bioscience version 2022.2.1). The number of Nissl-stained neurons was estimated using the optical fractionator method as described previously.^22^ The dissector height was 13.0 μm, which was approximately the total section thickness as determined within StereoInvestigator and can be attributed to standard tissue shrinkage throughout processing. Counting frames (100 X 100 μm) were spaced randomly and systematically at intervals of 308 X 184 μm. The hilus was defined by the border with the granule cell layer and with a line drawn from the tip of the granule cell layer around the proximal tip of the cornu ammonis (CA)-3 pyramidal cell layer (Figure 1). The same investigator performed all counting to avoid any relative variability between dogs.

**Figure 2—:**
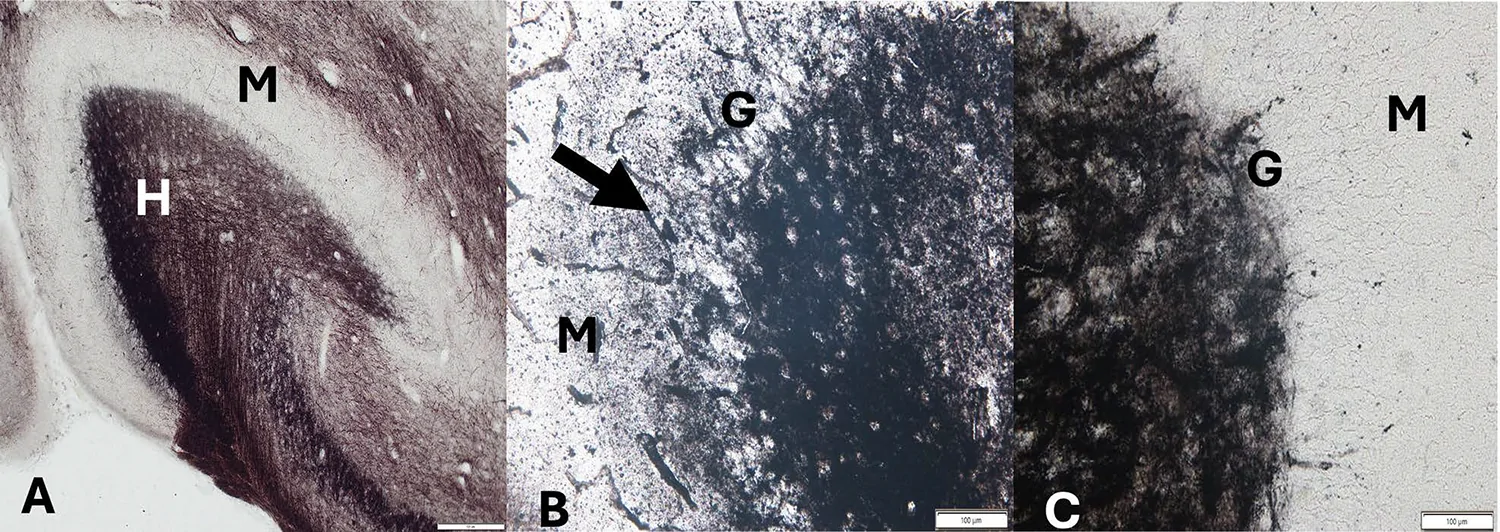
Timm-stained hippocampus at (A) 40X magnification, scale bar = 500 μm, (B) 200X magnification with mossy fiber sprouting scored at a 3 by all investigators, scale bar = 100 μm, and (C) 200X magnification with mossy fiber sprouting scored at a 0 by all investigators, scale bar = 100 μm. The arrow indicates mossy fiber sprouting.

After Timm staining was performed, 3 investigators (SC, SO, and PB) were masked to the dog being evaluated and gave a score from 0 to 5 of the extent of mossy fiber sprouting in 3 areas from 3 sections of each hippocampus, with 0 meaning there was no evidence of mossy fiber sprouting present and 5 meaning there was extensive mossy fiber sprouting present (Figure 2); therefore, 9 sections from each hippocampus were evaluated. The Timm staining protocol and the scoring system used have been described previously.^23^ All investigators underwent training, using a manual and presentations created by one of the authors (SO), an expert in mossy fiber sprouting, on what to look for when scoring as well as examples of each level from 0 to 5. Scores were then averaged between sections and investigators to result in an overall score for each hippocampus.

### Statistical analysis

Shapiro-Wilk testing for normality was performed, and if normally distributed, then 2 standard deviations above or below the mean were used to identify values significantly different from the mean of the entire sample. A *P* value of < .05 was considered statistically significant for all tests.

## Results

### Study population

Seven dogs with IE with tier 2 classification based on the International Veterinary Epilepsy Task Force^2^ with a history of focal epileptic seizures as their main semiology were included in the study and consisted of 2 females and 5 males. All dogs were humanely euthanized at the request of their owner due to worsening seizure progression (frequency and/or duration) despite appropriate antiseizure medications. In 2 dogs, the seizures generalized secondarily after starting as focal epileptic seizures. In general, seizure activity was described by a change in mentation, hypersalivation, and facial and/or body twitching with an accompanying postictal period. Ages at the time of euthanasia ranged from 3 to 12 years (median, 9 years). The following breeds were included: 4 mixed breeds, 1 Labrador Retriever, 1 Border Collie, and 1 Yorkshire Terrier. Weights ranged from 4.4 to 34.4 kg, with a median of 16.2 kg. Dogs with any other known or suspected intracranial disease (other than IE) were excluded from the study. Additional demographic data can be found in Table 1.

### Stereology analysis

**Figure 3—:**
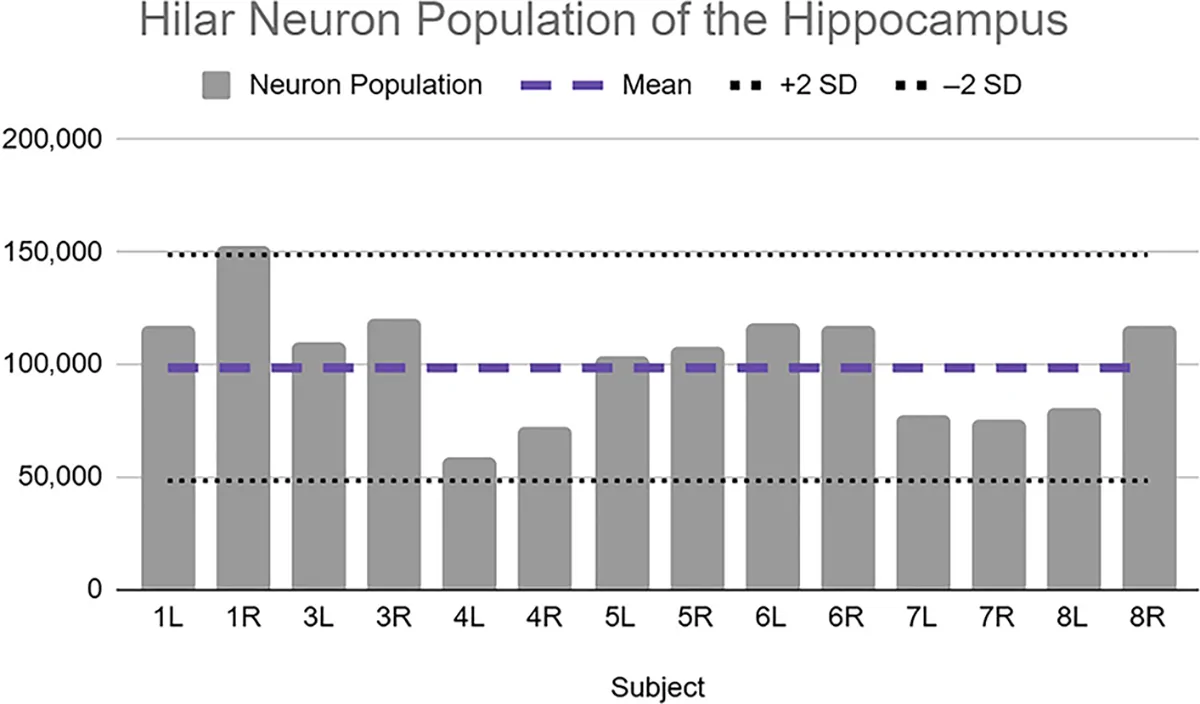
Nissl-stained hilar neuron population of each dog estimated via the optical fractionator method, with a range of 56,900 to 150,000 neurons and a median of 104,000 neurons. L = Left. R = Right.

Both the left and right hippocampi from each dog were analyzed. All stereological data are shown in Table 2. The average number of sampling sites per dentate gyrus within each hippocampus was 1,490. Only neuronal nuclei that were not cut at the top of the section were counted, and the average total number of neuronal nuclei counted per dentate gyrus was 610. Stereologic cell counting of Nissl-stained hilar neurons in the dentate gyrus resulted in a range for each hippocampus from 56,900 to 150,000 neurons, with a median of 104,000 neurons (Figure 3). To investigate the possibility of asymmetrical hippocampi, the hippocampus from each dog (either right or left) with the lower cell count was grouped and compared to the group of hippocampi with higher cell counts. No evidence of overall asymmetry was seen (*P* = .259). The mean of the stereological cell counts was 98,600 neurons, with an SD of 25,100. The sample of hilar neuron numbers was normally distributed (*P* = .27, Shapiro-Wilk test). One hippocampus (right; dog 1) had significantly more hilar neurons (150,000; Figure 3). No values were significantly lower than the mean.

### Timm analysis

**Figure 4—:**
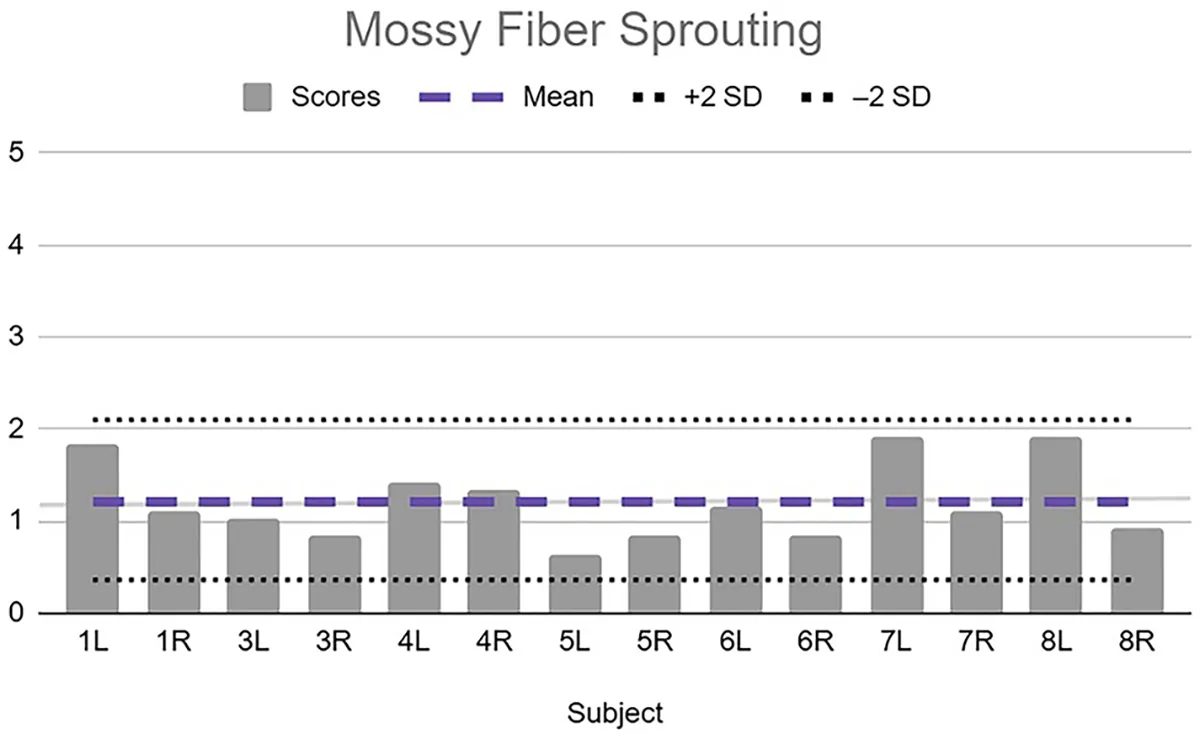
Timm-stained mossy fiber sprouting scores of each dog with a median of 1.11 (range, 0.63 to 1.93).

For each Timm-stained hippocampus (left and right), 3 regions from 3 different slides were reviewed. A score from 0 to 5 was given, with 0 meaning no evidence of mossy fiber sprouting into the supragranular layer and a score of 5 indicating extensive and severe mossy fiber sprouting into the supragranular layer. Average scores for Timm staining for each hippocampus ranged from 0.63 to 1.93, with a median of 1.11 for all dogs (Figure 4). The sample of Timm stain scores was normally distributed (*P* = .078, Shapiro-Wilk test). To investigate the possibility of asymmetrical hippocampi, the hippocampus with the lower cell count (based on stereology) from each dog was grouped together and compared to the group of hippocampi with higher cell counts based on stereology. No significant evidence of asymmetry (*P* = .054) was found. The mean of the Timm stain scores was 1.21, with an SD of 0.423. No values were significantly different than the mean Timm stain score.

## Discussion

Our study found no evidence of asymmetry between hippocampi in either hilar neuron counts or Timm stain scores, no significant hilar neuron cell loss in any hippocampus, and mild mossy fiber sprouting with no significant differences from the mean score. The lack of neuropathological evidence of TLE in dogs with focal epileptic seizures is consistent with the findings of a previous study^15^ in dogs with IE and generalized seizures.

We estimated an average of 98,600 hilar neurons/CA4 region of the hippocampus. This falls between what has been found previously for rats (53,000)^22^ and what has been found for cats (107,000),^24^ dogs with medically intractable IE (186,000),^15^ sea lions (333,000),^19^ and humans (2,000,000).^25^ The difference between previous findings in epileptic dogs and our study might be attributable to differences in subject populations. Our study population included older patients, and some hilar neuron loss occurs within the hippocampus with aging.^26^ A previous study^27^ found a 30% loss of hilar neurons in aged (13 to 15 years old), unenriched subjects compared to young (3.4 to 4.5 years old) subjects.

In humans with TLE^8^ and in rodents with experimentally induced TLE,^28^ there is extensive hilar neuron loss and robust mossy fiber sprouting. Within our cohort, there were no hippocampi that had a hilar neuron cell count less than twice the SD of the study group as a whole, none with mossy fiber sprouting exceeding twice the SD of the study group, and no significant asymmetry between hippocampi in either neuronal cell counts of mossy fiber sprouting. While there is evidence of hippocampal asymmetry in a subset of dogs with IE demonstrated via MRI volumetric analysis,^16,29^ seizure activity can result in neuronal injury and subsequent neuronal loss.^8^ Focal seizures have a seizure focus in only 1 hemisphere,^30^ so the hippocampal asymmetry found could be solely a result of focal seizures due to any cause and not specifically related to TLE.

The majority of cases (60% to 80%) of human TLE-HS are International League Against Epilepsy type 1, meaning that both CA1 and CA4 are most severely affected by neuronal cell loss; however, all hippocampal subareas have significant neuronal cell loss.^31^ It is possible that the patterns of TLE-HS present differently in dogs and may be more localized to specific subareas, so future directions could include analyzing additional sections of the hippocampus in search of neuronal cell loss.

The lack of a control group is a limitation of this study as only within-group analysis can be performed rather than comparison to a cohort without seizures. Additionally, it is possible that even though our study sample had a history of focal epileptic seizures, TLE may still be present in a subset of dogs with epilepsy—just not our study sample. Future directions could include a larger sample size of dogs with focal epileptic seizures or more specific neuropathological testing in dogs that have antemortem diagnostics (ie, MRI) that reveal asymmetric hippocampi.

In conclusion, the mild amount of mossy fiber sprouting and lack of significant hilar neuron loss, as well as the lack of significant asymmetry between hippocampi, suggest that this cohort of dogs with IE and focal epileptic seizures does not have TLE.

## Figures and Tables

**Table 1—:** Demographics of 7 dogs within this study.

Dog ID No.	Breed	Age at euthanasia (y)	Weight (kg)	Sex	Seizure type

1	Labrador Retriever	8.1	34.3	F, S	Focal
3	Golden Doodle	8.9	21.2	M, N	Focal
4	Poodle mix	12	5.9	M, N	Focal
5	Bichon mix	9	4.4	F, S	Focal
6	Border Collie	11	16.8	M, N	Focal onset, secondary generalization
7	Yorkshire Terrier	4	7.5	M, N	Focal
8	Pit Bull Terrier	2.9	23	M, N	Focal onset, secondary generalization

F = Female. M = Male. N = Neutered. S = Spayed.

**Table 2—:** Parameters and results of stereologic counting for Nissl-stained neurons.

Subject	Total markers counted	No. of sections	No. of sampling sites	Estimated population using mean section thickness with counts	Coefficient of error (Gundersen)

1L	680	13	1,714	112,674	0.04
1R	908	19	1,735	150,130	0.03
3L	666	19	1,967	107,401	0.04
3R	733	19	1,801	118,282	0.04
4L	368	10	917	56,865	0.06
4R	411	10	1,046	69,250	0.05
5L	663	11	1,182	100,848	0.04
5R	616	11	1,311	100,222	0.04
6L	721	11	1,731	114,206	0.04
6R	715	14	2,104	113,408	0.04
7L	435	10	1,012	75,496	0.05
7R	440	11	1,194	71,860	0.05
8L	488	9	1,341	78,631	0.05
8R	694	12	1,848	111,867	0.04

L = Left hippocampus. R = Right hippocampus.
